# Thermally Stabilised Poly(vinyl alcohol) Nanofibrous Materials Produced by Scalable Electrospinning: Applications in Tissue Engineering

**DOI:** 10.3390/polym16142079

**Published:** 2024-07-21

**Authors:** W. Joseph A. Homer, Maxim Lisnenko, Sarka Hauzerova, Bohdana Heczkova, Adrian C. Gardner, Eva K. Kostakova, Paul D. Topham, Vera Jencova, Eirini Theodosiou

**Affiliations:** 1 Engineering for Health Research Centre, College of Engineering and Physical Sciences, Aston University, Birmingham B4 7ET, UK; w.homer1@aston.ac.uk; 2 Chemical Engineering and Applied Chemistry, College of Engineering and Physical Sciences, Aston University, Birmingham B4 7ET, UK; p.d.topham@aston.ac.uk; 3 Department of Chemistry, Faculty of Science, Humanities and Education, Technical University of Liberec, 461 17 Liberec, Czech Republic; maxim.lisnenko@tul.cz (M.L.); sarka.hauzerova@tul.cz (S.H.); eva.kostakova@tul.cz (E.K.K.); vera.jencova@tul.cz (V.J.); 4 Department of Haematology, Regional Hospital Liberec, 460 01 Liberec, Czech Republic; bohdana.heczkova@nemlib.cz; 5 The Royal Orthopaedic Hospital NHS Foundation Trust, Birmingham B31 2AP, UK; adrian.gardner@nhs.net; 6 College of Health and Life Sciences, Aston University, Birmingham B4 7ET, UK; 7 Aston Advanced Materials Research Centre, Aston University, Birmingham B4 7ET, UK

**Keywords:** non-woven fibres, electrospinning, poly(vinyl alcohol), cross-linking, tissue engineering

## Abstract

Electrospinning is a widely employed manufacturing platform for tissue engineering applications because it produces structures that closely mimic the extracellular matrix. Herein, we demonstrate the potential of poly(vinyl alcohol) (PVA) electrospun nanofibers as scaffolds for tissue engineering. Nanofibers were created by needleless direct current electrospinning from PVA with two different degrees of hydrolysis (DH), namely 98% and 99% and subsequently heat treated at 180 °C for up to 16 h to render them insoluble in aqueous environments without the use of toxic cross-linking agents. Despite the small differences in the PVA chemical structure, the changes in the material properties were substantial. The higher degree of hydrolysis resulted in non-woven supports with thinner fibres (285 ± 81 nm c.f. 399 ± 153 nm) that were mechanically stronger by 62% (±11%) and almost twice as more crystalline than those from 98% hydrolysed PVA. Although prolonged heat treatment (16 h) did not influence fibre morphology, it reduced the crystallinity and tensile strength for both sets of materials. All samples demonstrated a lack or very low degree of haemolysis (<5%), and there were no notable changes in their anticoagulant activity (≤3%). Thrombus formation, on the other hand, increased by 82% (±18%) for the 98% hydrolysed samples and by 71% (±10%) for the 99% hydrolysed samples, with heat treatment up to 16 h, as a direct consequence of the preservation of the fibrous morphology. 3T3 mouse fibroblasts showed the best proliferation on scaffolds that were thermally stabilised for 4 and 8 h. Overall these scaffolds show potential as ‘greener’ alternatives to other electrospun tissue engineering materials, especially in cases where they may be used as delivery vectors for heat tolerant additives.

## 1. Introduction

Tissue engineering (TE) combines the ingenuity and problem-solving of engineering with the fields of medicine and biology to develop substitute technologies and methods capable of maintaining, restoring, and enhancing tissue function beyond what has been historically possible through more traditional or natural means. Commonly, TE utilises regenerative cell treatments (scaffold-free methods), and advanced biomaterials (scaffold-based methods) to promote tissue recovery around a targeted defect site [1]. The materials developed for scaffold-based TE span a broad range of production methods, including solvent casting, 3D printing, freeze-drying and electrospinning, to name a few [2,3]. These can produce quite distinct material morphologies with differing properties, and while some properties, such as biocompatibility and capacity for diffusion of nutrients, are typically a ubiquitous requirement for all TE materials, others, such as mechanical strength and capacity for drug loading, can vary depending on application [4]. 

Among the many manufacturing techniques used, electrospinning has been explored extensively within TE [5,6]. Electrospinning relies on the application of electrical forces to transform solutions into a nanofibrous material and is driven either by direct current (DC electrospinning) or alternating current (AC electrospinning [7]). DC electrospinning utilises electrostatic forces by applying a charge to a polymer solution, accelerating it towards oppositely, or neutrally charged collectors, resulting in the formation of fine liquid jets with diameters in the micro- to nanometre range. Major benefits of electrospinning include the fact it is a straightforward process that can be set up for very low costs, can produce nanofibers with tuneable sizes, and has the ability to control fibre orientation, with a high degree of reproducibility [2,8,9,10]. Equipment for scalable electrospinning has also seen commercial success in more recent years, with the development of a number of industrial-scale systems [11,12]. The final fibrous morphology of the electrospun materials can frequently mimic the extracellular matrix (ECM), thereby offering a good substrate for cell proliferation, while the highly interconnected pores allow for easy diffusion of nutrients, and enhanced loading efficiency for drug delivery [13]. Electrospinning is especially popular for the creation of active wound dressings, with recent examples including nanofibrous hydrogels containing antimicrobial agents, as well as the use of alternative current for enhanced productivity [7,14]. Fibres for TE have been produced from a range of materials, with some common examples including collagen, polycaprolactone, poly(lactic acid), silk fibroin, and poly(vinyl alcohol) (PVA) [8,15,16]. 

**Figure 1:**
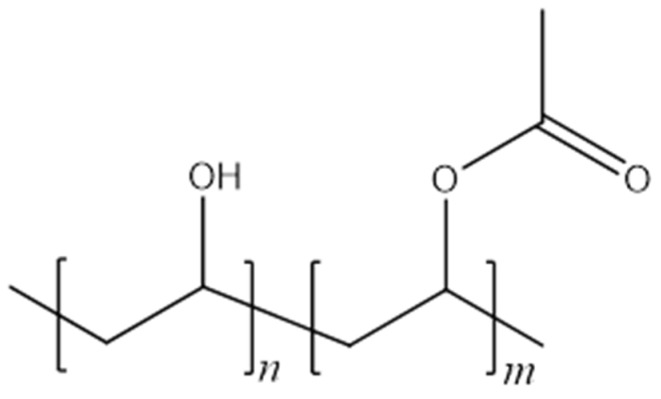
Chemical structure of PVA, where the left monomer unit is PVA, at a relative molar abundance of *n*%, and the right monomer unit shows a residual acetate group of relative molar abundance of *m*%.

Among others, PVA is quite popular for a range of biomedical applications, including bone, skin, vascular, and corneal TE [16]. It is a low-cost hydrophilic polymer, typically derived by the hydrolysis of poly(vinyl acetate), where the percentage conversion of acetate groups into hydroxyls defines the degree of hydrolysis (DH) (Figure 1). It is considered non-toxic and biocompatible and therefore has received FDA approval for food packaging and biomedical applications [17,18,19]. PVA is readily water-soluble, with the rate of dissolution in aqueous environments being dependent upon its degree of hydrolysis (DH) and molecular weight. This enhanced solubility can be perceived as a disadvantage for most applications, and therefore, it is not uncommon for PVA (either in hydrogel or fibrous formats) to be routinely combined with chemical crosslinkers, such as glutaraldehyde (GA) or glyoxal to reduce water uptake. However, these chemicals are typically toxic, and the presence of any residual cross-linkers can be considered problematic [20,21]. For certain applications, such as skin or bone TE, it can be highly desirable to match the rate of material degradation to the rate of healing but also tailor the mechanical properties of the scaffold to the tissue type (e.g., hard vs. soft tissue), function and location in the body [22,23,24]. In those cases, the advantages of using PVA that has been cross-linked without the use of toxic chemicals, are apparent since the final scaffold could possess tailor-made properties that can be adjusted according to the degree of hydrolysis and treatment duration [25]. 

Herein, thermal treatment at 180 °C has been studied for two highly hydrolysed (98% and 99%) PVA nanofibrous fabrics, in the absence of toxic cross-linkers. Heating is believed to stabilise the morphology of the PVA fibres in aqueous environments, through the enhancement of material crystallinity (physical cross-linking) and possibly the creation of chemical cross-links in the form of ether bonds [25]. The physicochemical implications of varying the degree of hydrolysis and the heating duration are considered in the context of TE, via material biocompatibility and hemocompatibility testing, to assess their potential use in vivo for different applications.

## 2. Materials and Methods

### 2.1. Materials

Poly(vinyl alcohol) (PVA) ‘Mowiol 20-98’ (98% hydrolysed, molecular weight (Mn) of 125,000 g·mol^−1^), PVA ‘Mowiol 28-99’ (99% hydrolysed, Mn of 145,000 g·mol^−1^), and phosphate-buffered saline (PBS, pH 7.4) were purchased from Sigma-Aldrich (St. Louis, MO, USA). Ethanol (≥99.8%) and GA (50%) were obtained from Fisher Scientific (Zurich, Switzerland). Composol PS solution for the preservation of thrombocytes on thrombocyte-rich solution (TRS)-exposed samples was acquired from Fresenius Kabi (Bad Homburg, Germany). Mouse fibroblasts (3T3) for biocompatibility testing were purchased from ATCC (Manassas, VA, USA) and cultured using Dulbecco’s Modified Eagle Medium (DMEM), with 10% FBS, and Glutamine supplied by Biosera (Nuaillé, France) and Penicillin-Streptomycin-Amphotericin B antibiotic mixture supplied by Lonza (Basel, Switzerland), while the assay carried out using cell counting kit-8 (CCK-8) was bought from Abcam (Cambridge, UK). Samples were sterilised using an Anprolene AN74i Ethylene-oxide cabinet (Andersen Sterilizers, Clacton-On-Sea, UK). Human blood was collected from healthy donors with informed consent in vacutainers containing 0.129 M sodium citrate obtained from BD (Franklin Lakes, NJ, USA). All chemicals were of analytical grade (unless otherwise stated) and used without further purification.

### 2.2. Production and Stabilisation of Electrospun Materials

Materials were produced using needleless direct current (DC) electrospinning from solutions of 98% and 99% hydrolysed PVA as described in previous work [25,26,27]. Briefly, 98% and 99% solutions were dissolved in 9:1 (*w*/*w*%) water/ethanol mixtures at polymer concentrations of 10% and 8%, respectively, and electrospun onto a polypropylene substrate moving at 20 mm·min^−1^ using an NS1S500U electrospinning rig (Elmarco, Liberec, Czech Republic), with a 50 kV electrode differential, electrode separation distance of 16 cm, and with atmospheric conditions held at 22 °C and 30% relative humidity by NS AC150 (Elmarco, Liberec, Czech Republic). These samples were removed from their substrate and subjected to temperatures of 180 °C for durations of 0, 1, 4, 8 and 16 h in a forced convection drying oven (SciQuip Oven-80 HT; Newtown, UK) before being allowed to cool at room temperature.

### 2.3. Analytical Methods

#### 2.3.1. Scanning Electron Microscopy

Scanning electron microscopy (SEM) was used to examine the morphology of samples and measure the effect of heat treatment on fibre diameters. Samples were prepared for analysis by sputter-coating with gold using a Quorum (Q150R ES) sputter coater and then imaged using a Tescan Vega3 SB Easy Probe (Tescan, Brno, Czech Republic). Subsequently, images were analysed using ImageJ software v1.52a (NIH, Bethesda, MD, USA).

#### 2.3.2. Fourier Transform-Infrared Spectroscopy

Fourier transform-infrared spectroscopy (FT-IR) was performed using a Frontier Spectrometer (PerkinElmer Ltd., Waltham, MA, USA) combined with an ATR accessory (GladiATR; Pike Technologies, Madison, WI, USA), using a scan range from 4000 cm^−1^ to 700 cm^−1^ with a resolution of 4 cm^−1^ for a total of 16 scans per measurement.

#### 2.3.3. X-ray Diffraction

Material crystallinity was evaluated using X-ray diffraction (XRD) on a Bruker D8 Advance diffractometer, equipped with a LynxeyePSD detector (Bruker, Billerica, MA, USA) and with Cu Kα1,2 radiation (40 kV and 40 mA), 0.02 mm Ni Kβ absorber, 5–50° 2θ range, a step scan of 0.02° with a sample rotation speed of 30 RPM. The degree of crystallinity was calculated using Equation (1), where α is the degree of crystallinity, *I_c_* is the sum of the intensity under the crystalline peaks, and *I_a_* is the sum of the intensity under the amorphous sections of the spectra.

(1) $$\alpha \left(\%\right)=\frac{I_{c}}{I_{c}+I_{a}}*100$$

#### 2.3.4. Tensile Testing

The mechanical properties of the mats were recorded by means of uniaxial tensile tests using an Instron 5965 (Instron, High Wycombe, Buckinghamshire, UK), equipped with a 50 N load cell, moving at a rate of 10 mm·min^−1^. Samples were prepared using a punch tool in a dumbbell shape (21 µm average thickness, n = 3) and continuously loaded until failure (defined as a 50% reduction from peak force). Stress–strain curves were obtained and mean averages of the UTS and strain at UTS were calculated.

#### 2.3.5. Contact Angle

Contact angle experiments were performed to examine changes in the hydrophilicity of the material because of the duration of thermal treatment. Due to limitations of wicking behaviour with electrospun samples, 15 mm diameter disc films were prepared by filling a silicon mould with the same solutions used in the electrospinning process, and the moulds were left to allow the films to form in a drying cabinet maintained at 25 °C for 2 days until they solidified. These films were then subjected to the same treatment durations of 1, 4, 8 and 16 h at 180 °C as the electrospun non-woven mats. Contact angle testing was performed using an Attension Theta Flex to carry out sessile drop testing and analysed using OneAttension software v4.0.3 (r8310) (Biolin Scientific, Gothenburg, Sweden). The liquid used was DI water, with a droplet volume of 5 μL and recording duration of 10 s following liquid–sample contact. 

#### 2.3.6. Biocompatibility Testing

Samples were prepared as 13 mm diameter discs to match the dimensions of the base of 24-well plates and sterilised by ethylene-oxide treatment in an Anprolene AN74i cabinet (Andersen Sterilizers, Haw River, NC, USA). Well plates with inserted discs were then seeded with 10^4^ 3T3 murine fibroblasts per well on day 0. Plates were incubated at 37 °C in the presence of 5% CO_2_, and CCK-8 assays were carried out on days 1, 3 and 7. Cell culture media was composed of DMEM with the addition of 10% FBS, 1% glutamine and 1% antibiotic mixture (Penicillin-Streptomycin-Amphotericin B). For each time point measured, media from each well was aspirated and exchanged with 500 μL of complete media containing 10% CCK-8, which was then incubated for 3 h before final absorbance values were measured at 450 nm using a BioTek Synergy HTX (Agilent Technologies, Inc., Santa Clara, CA, USA) (n = 4). After the final time point on day 7, cells were fixed with 2.5% GA solution in PBS for 30 min at 4 °C, then washed with a series of ethanol solutions with increasing concentration (60%, 70%, 80%, 90%, 95%, 100%) before being prepared for SEM as per Section 2.3.1.

#### 2.3.7. Hemocompatibility—Collection and Preparation of Blood Product Solutions

Preparation of blood products for hemocompatibility testing was carried out according to Horakova et al. [28]. Routinely, whole blood for haemolysis assays was collected from healthy donors in 4 mL BD vacutainers containing 0.129 M sodium citrate and then diluted with 5 mL PBS. Coagulation testing was carried out using clinical plasma provided by Liberec Regional Hospital and collected with informed consent from donors. TRS for thrombogenicity testing was prepared by combining buffy coats collected from 4 blood donors (collected by the Liberec Regional Hospital with informed consent) after centrifugation using a deleukocytation filter (CompoStop Flex 3F T&B, Fresenius Kabi, Bad Homburg, Germany).

##### Thrombogenicity

Thrombogenicity was tested by incubating the materials at 37 °C in the presence of TRS for 2 h and subsequently analysing each material using CCK-8 assay and SEM imaging. Nanofibrous mats were sterilised using ethylene-oxide, prepared in 6 mm discs, and placed in the wells of a 96-well plate. 200 µL of TRS (concentration 7.85 × 10^6^ thrombocytes per mL), was placed in each well, and after 2 h of incubation, the TRS was aspirated, and the samples were washed twice with PBS. Materials for SEM analysis were fixed using 2.5% GA in PBS solution for 30 min at 4 °C, followed by rinsing with a series of graded ethanol solutions (60–100%). For thrombocyte viability testing, culture media was exchanged with fresh Intersol PS containing 10% CCK-8 for 3 h. Final absorbance values were measured at 450 nm using a Spark multimode microplate reader (TECAN Spectrophotometer, Grödig, Austria) (n = 10).

##### Coagulation

The clotting time of clinical plasma was measured after incubation with all electrospun materials to determine the prothrombin time (PT) and activated partial thromboplastin time (APTT). Samples were prepared as 1 cm^2^ swatches, placed in test tubes and combined with 300 μL of clinical plasma. For a negative control, the plasma was incubated in the absence of any materials. Test tubes were incubated for 45 min at 37 °C before samples were removed from the solution. Clotting time was measured using an automatic BCS XP analyser (Siemens, Munich, Germany) according to the manufacturer’s instructions (n = 5).

##### Haemolysis

As for coagulation experiments, 1 cm^2^ swatches were prepared from nanofibrous membranes, which were then added to test tubes containing 10 mL of PBS. Negative and positive control tubes contained 10 mL of PBS and distilled water, respectively, in the absence of any materials. All samples were incubated at 37 °C for 30 min, prior to dosing with 200 µL of anticoagulated diluted whole blood prepared earlier, and incubated for a further 60 min. The tubes were then centrifuged at 100× *g* for 5 min, the supernatant was aspirated from the container into well plates, and the absorbance was measured at 570 nm using a Spark multimode microplate reader (TECAN Spectrophotometer, Austria) (n = 5, 2 measurements per sample).

## 3. Results and Discussion

Nanofibrous non-woven supports were produced by needleless DC electrospinning using 98% and 99% hydrolysed PVA and thermally stabilised for 1, 4, 8 and 16 h at 180 °C. The supports were then compared to assess the effect of the degree of hydrolysis and heat treatment duration on their potential use as biomaterials in TE applications. First, the samples were evaluated based on their morphological, physicochemical, and mechanical properties before investigating their biocompatible and haemocompatible properties, which are essential for TE materials.

### 3.1. Morphological Comparison of Nanofibers Produced from 98% and 99% Hydrolysed PVA

**Figure 2:**
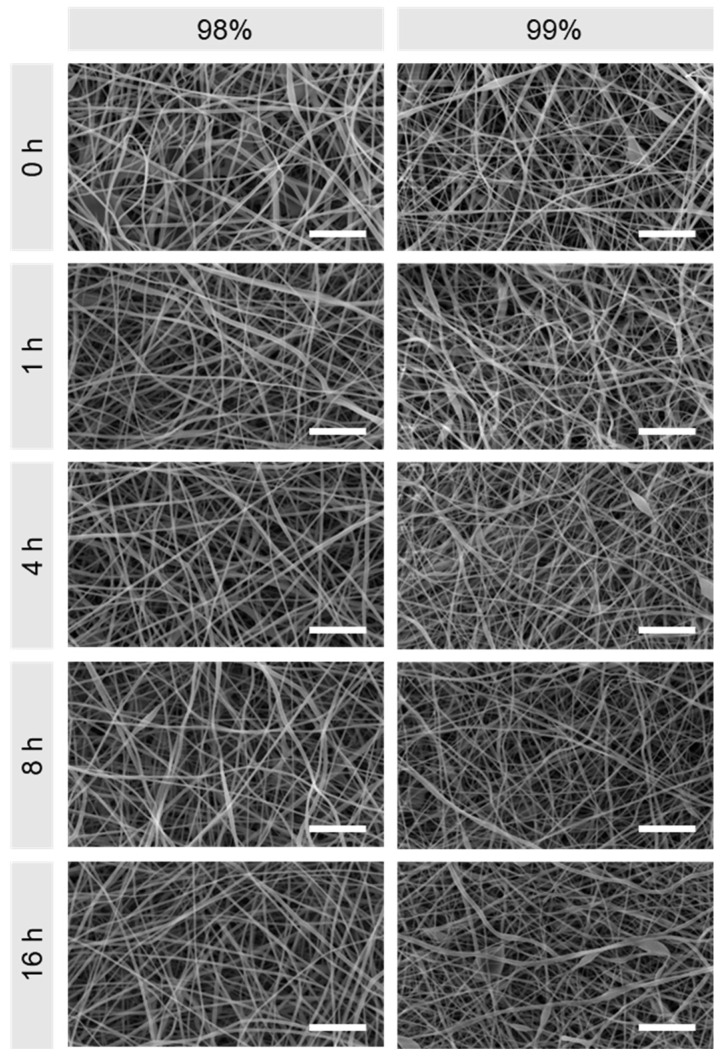
SEM images of 98% and 99% hydrolysed electrospun PVA following heat treatment at 180 °C for 0, 1, 4, 8 and 16 h. Scale bars: 10 µm.

The nanofibrous structure of electrospun materials can be viewed as one of the closest morphological mimics to the ECM [29] and therefore a contributing factor in achieving enhanced cell proliferation in vitro during tissue regeneration [30,31]. Figure 2 shows SEM images of 98% and 99% hydrolysed PVA for the respective heat treatment durations. It is apparent that, when untreated, both materials present highly fibrous structures with few defects, and this remains unchanged following heat treatment. 

**Figure 3:**
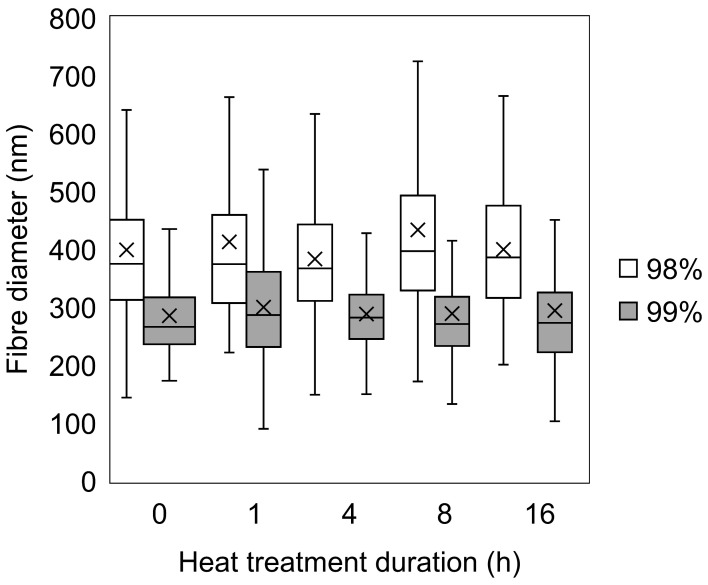
Box and whisker plot of fibre diameters for 98% and 99% hydrolysed PVA needleless electrospun mats heat treated for 0–16 h. Key: 98% (white); 99% (grey). Full histograms are available in Figure S1.

The degree of hydrolysis appears to have some influence on the size of the fibres (Figure 3). For untreated samples, the average fibre diameter for 98% hydrolysed PVA samples was 399 nm (±153 nm, CI = 95%, n = 100), compared to 285 nm (±81 nm, CI = 95%, n =100) for the 99% hydrolysed sample. The average fibre diameter for 98% hydrolysed PVA across all heat-treated samples measured was 406 nm, ranging from 383 nm (4 h) to 434 nm (8 h), whilst the average diameters for 99% samples was 291 nm, ranging from 285 nm (0 h) to 300 nm (1 h). These findings are in agreement with Park and co-workers [32] who documented an exponential increase in diameters with DH between 88% and 99.9%. Finally, and although it seems that heat-treated fibres appear marginally thicker, the observed difference in averages was not considered notable relative to the standard deviation of the samples, thereby suggesting that heat treatment does not markedly alter fibre morphology. 

### 3.2. Physicochemical Comparison of Nanofibers Produced from 98% and 99% Hydrolysed PVA

#### 3.2.1. Fourier-Transform Infrared Spectroscopy

**Figure 4:**
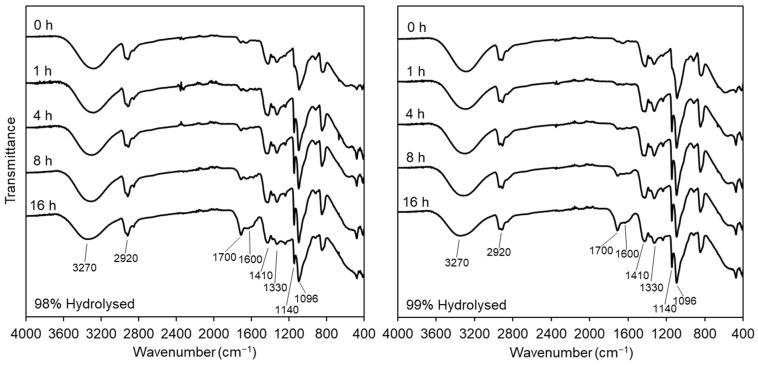
FT-IR Spectra of two sample series of 98% (**left**) and 99% (**right**) DH PVA produced by needleless electrospinning with heat treatment time points indicated on the offset spectra.

The key characteristic peaks of the FT-IR spectra of PVA are briefly summarised from the literature in Table S1, including key C-O stretching at 1140 cm^−1^ and O-H bending at 1096 cm^−1^ commonly associated with crystalline and amorphous phases, respectively [33,34,35]. Given the small difference of only 1% residual acetate groups between the hydrolysis percentage of the PVA samples, it was not anticipated that there would be any significant differences between untreated nanofibrous samples, which was confirmed by the 0 h spectra in Figure 4. As observed in previous work, heat-treated samples of all durations produced higher absorption of the 1140 cm^−1^ peak, attributed to a combination of C-O stretching due to both increases in crystallinity and in the prevalence of C-O-C bonds [25]. At this signal along with the broad O-H signal at 3270 cm^−1^, we see a slight reduction in absorbance in the samples treated for 16 h, which could be associated with reduced crystallinity (discussed further in Section 3.2.2). As heat treatment progresses, the increase in peaks at 1600 and 1700 cm^−1^ is most likely attributed to C=C and C=O bonds, respectively, due to potential thermolysis [36,37]. Degradation of PVA from the literature identifies a number of routes, including chain scission, dehydration, and intermolecular dehydration, resulting in chemical crosslinking, all of which appear to be present within the materials. This, however, poses the question of the safety and suitability of partially degraded materials for biological applications. Therefore, bio- and hemocompatibility testing were used to indicate to what degree this degradation is tolerated in a TE context and are discussed in Section 3.3.

#### 3.2.2. X-ray Diffraction

**Figure 5:**
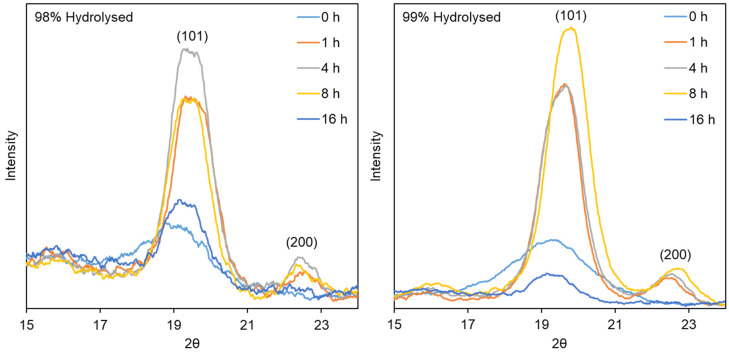
XRD spectra of 98% (**left**) and 99% (**right**) DH PVA materials produced by DC electrospinning. Key: 0 h (light blue); 1 h (orange); 4 h (grey); 8 h (yellow); 16 h (dark blue).

All samples were analysed using XRD to compare their crystallinity, both in terms of differences between species of PVA, as well as arising from heat treatment duration (Figure 5). The monoclinic unit cell reflections shown at (101) and (200) suggest an increase in polymer chain ordering for samples treated for 1, 4 and 8 h in both species, followed by a sharp reduction at 16 h, as suggested by the FT-IR results. As the heat treatment temperature increases, the strength of intermolecular interactions between PVA chains mediated by hydrogen bonds, increases. After the preparation of PVA nanofibers, certain residual water molecules remain in the material’s structure [38]. As a result of heat treatment, the number of PVA segments with a high degree of hydration is reduced; at the same time, as the temperature increases, the mobility of the polymer chains also increases, which facilitates the formation of the crystalline phase of the polymer. The crystalline regions serve as a source of strong physical cross-linking due to the hydrogen bridges formed between the hydroxyl groups of the PVA chains, which maintain the material’s structure in an aqueous environment [39].

Numerical data were generated using Equation (1), and as can be seen in Table 1, crystallinity fell from 26.8% to 19.5% for 98% DH samples, and from 57.0% to 19.5% for 99% DH samples. The samples produced from 99% DH PVA are consistently more crystalline than their 98% DH counterparts, and although this is not surprising, based on the reduction in the steric effect produced by halving the residual acetate groups, it was more pronounced than expected. A possible explanation could be the limitation of applying this equation to materials, such as those made from polymers, which have only partial ordering but also are relatively thin in nature. This may well result in baseline signals (i.e., the area under the amorphous region of the spectra, *I_a_*), which are not strictly reliable. Nevertheless, it can still be used cautiously as a tool for relative comparison within a set of data, such as the heat treatment series presented here, to indicate the increase and subsequent decrease in crystallinity within each series separately.

#### 3.2.3. Tensile Testing

**Figure 6:**
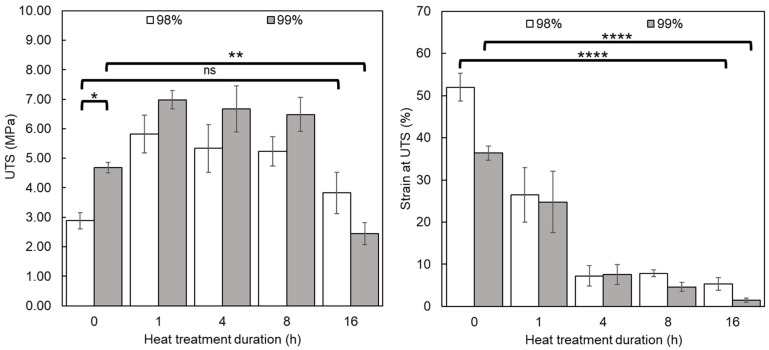
Tensile performance of samples produced from 98% and 99% hydrolysed PVA electrospun mats after thermal stabilisation. (n = 3). Key: 98% (white); 99% (grey). Representative stress–strain curves can be found in Figure S2. ns *p* > 0.05, * *p* ≤ 0.05, ** *p* ≤ 0.01, **** *p* ≤ 0.0001.

Tensile load testing was carried out to assess the UTS and displacement at UTS of the nanofiber mats following heat treatment and compare samples produced from PVA with differing DH (Figure 6). The supports that were electrospun from 99% DH PVA appeared significantly stronger than those created from 98% DH PVA (*p* = 0.0032), with untreated samples having 62% greater UTS (4.68 MPa for 99% DH c.f. 2.88 MPa for 98% DH). This is most likely due to the reduced chain mobility as a result of fewer acetate groups and increased hydrogen bonding. A similar trend was also observed within a polymer blend by Restrepo et al. [40], where it was shown that PVA with a higher degree of hydrolysis resulted in stronger materials. Strength differences in heat-treated mats were 20–25% greater in the 99% DH sample than the equivalent 98% samples, with the exception of the 16 h treated sample, which was ~36% weaker. A lower degree of hydrolysis samples (98%) achieved UTS of 5.82 (1 h), 5.33 (4 h), 5.23 (8 h), and 3.82 MPa (16 h) compared to UTS of 6.98, 6.67, 6.48, and 2.45 MPa for the respective pairs from 99% hydrolysed PVA. This too is attributed to the lack of steric hindrance caused by a decrease in the large acetate groups in the 99% hydrolysed PVA and the formation of strong hydrogen intramolecular bonds leading to increased ordering of the polymer chains and more effective chain packing [41]. The results clearly demonstrate increased strength at the first treatment time point, caused by the enhanced crystallinity of PVA by the annealing process, followed by the steady reduction in material strength due to thermal degradation identified by FT-IR. Young’s modulus was also seen to be higher in all 99% hydrolysed samples (30, 145, 158, 125, and 82 MPa for 98% samples treated for 0–16 h, respectively, and 207, 311, 245, 238, and 167 MPa for 99% hydrolysed samples). Untreated materials were 6.9 times stiffer, most likely due to the aforementioned enhanced intermolecular bonding. Annealing then enhanced the modulus of the samples treated for 1–16 h, resulting in samples produced from 99% DH PVA, which were on average 85% stiffer than their 98% counterparts.

Conversely, and due to differences in chain mobility, untreated 98% DH samples tolerated strain at UTS, which was on average 15.6% greater than the 99% DH untreated mats (*p* = 0.0144), while treated versions of both sample series performed more similarly across all durations (average strain at UTS of 25.7%, 7.4%, 6.2%, and 3.4% for 1 h, 4 h, 8 h and 16 h treatments, respectively). Ultimately, both samples reduced in ductility as treatment duration increased (*p* < 0.0001), becoming increasingly brittle. This could be understood as a combination of increased chemical crosslinking of some chains, thereby reducing elastic properties, whilst also increasing the extent of chain scission after 1 h, producing a progressively weaker material. 

Mechanical properties are an important parameter in TE, and depending on the application, biomaterials can be subject to a range of mechanical stresses and load patterns [42]. For example, while the demands of materials designed for bone TE may focus on material strength, desirable properties for neuronal TE may be more centred around rheological properties such as the storage modulus [43,44]. Therefore, it is not possible to point out which of the non-woven mats created in this work is better than the others in that respect, but we can conclude that this approach allows for better flexibility in the production of tailor-made biomimetic materials to suit a specific purpose. 

#### 3.2.4. Contact Angle

Initially, contact angle testing was carried out using PVA nanofibrous mats, but due to surface interactions between the water and the hydrophilic fibres, droplets on the surface of the material did not remain stable and effectively dissipated. Therefore, films were created from the same solution as the one used in the electrospinning process for each material series and heat-treated at the same time points as the nanofiber samples. 

**Figure 7:**
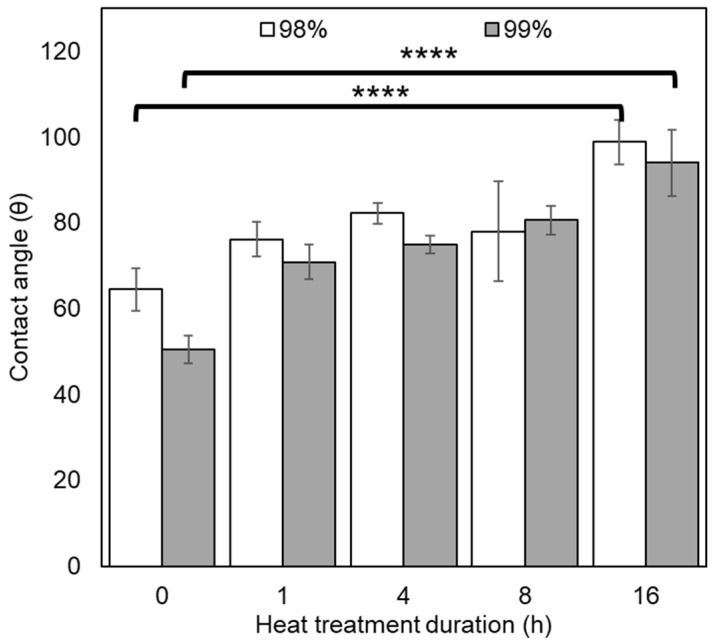
Sessile drop contact angle data for films produced from 98% and 99% hydrolysed PVA after thermal stabilisation. (n = 3). Key: 98% (white); 99% (grey). **** *p* ≤ 0.0001.

Sessile drop testing on these films indicated a significant difference in contact angle due to both DH (*p* = 0.0288) and heat treatment duration (*p* < 0.0001). It was found that the contact angle of 98% DH PVA was generally higher than their 99% counterparts, as shown in Figure 7, with corresponding optical images in Figure S3. This is also reflected in the literature, where PVA with higher DH produces lower contact angles with water—a trend which appears to remain post-heat treatment, suggesting that polar functional groups (i.e., hydroxyl groups) are more abundant in the 99% DH PVA films even after annealing [45]. Untreated films produced contact angles of 64.45° (98% DH) and 50.52° (99% DH), demonstrating their hydrophilic character, with the contact angle steadily rising until the materials developed increasing hydrophobic character (>90°) in the samples treated for 16 h (with values of 98.84° and 93.98°, for 98% and 99% DH, respectively). This is in line with expectations based on FT-IR results and the formation of polyenes due to thermolysis. In some instances, such as with some blood-contacting materials, increased hydrophobicity may be desirable for TE applications, where a mixture of hydrophobic and hydrophilic domains can improve compatibility, reducing protein adsorption and effects on coagulation [46].

### 3.3. Biological Assessment and Comparison of Nanofibers Produced from 98% and 99% Hydrolysed PVA

Following chemical, morphological, and mechanical characterisation, the samples were examined using a range of biological assessments, including bio- and hemocompatibility testing, to study the effect of heat treatment duration and degree of hydrolysis on cells. Though no meaningful change in fibre diameter was observed as a result of heat treatment, the chemical modifications and associated changes in surface contact angle and mechanical strength can influence cell–fibre interactions, with critical implications for tissue engineering applications [47,48]. These interactions were explored using combinations of colourimetric assays, SEM imaging and coagulation testing.

#### 3.3.1. Biocompatibility

**Figure 8:**
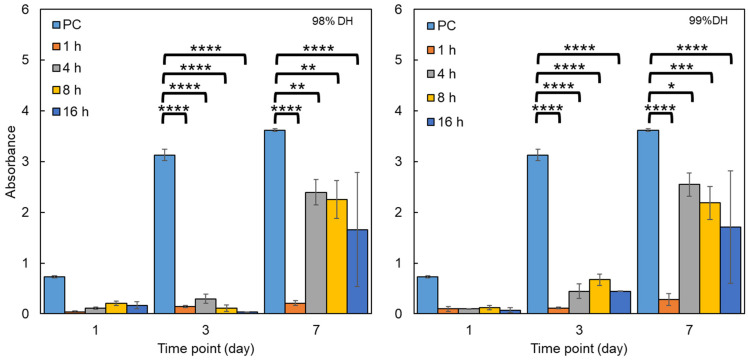
Cell viability results of 98% (left-hand side) and 99% (right-hand side) PVA nanofibrous mats after heat treatment at time points of 1, 3 and 7 days after seeding, with absorbance measured at 450 nm. Key: positive control/PC—well plate plastic (light blue); 1 h (orange); 4 h (grey); 8 h (yellow); 16 h (dark blue). (n = 4). * *p* ≤ 0.05, ** *p* ≤ 0.01, *** *p* ≤ 0.001, **** *p* ≤ 0.0001.

Biocompatibility tests were conducted using direct cytotoxicity testing by cultivating 3T3 mouse fibroblasts on the electrospun scaffolds and performing CCK-8 assays to assess relative cell viability. Cells were seeded (10^4^ cells per well) in 24 well plates on day 0, and cell viability assays were performed at 1, 3 and 7 days, measuring absorbance at 450 nm using a microplate reader (Figure 8). Untreated samples were excluded from the analysis as they dissolved immediately upon contact with cell culture media. On day 1, there was no notable difference in absorbance between wells containing nanofibrous mats, though the positive control in the empty well plate had much greater cell viability, indicating good adherence and early proliferation. 

On day 3, and following cell adaptation to the surface topography of the electrospun materials, some proliferation began to occur on both sample series. For the supports made from 98% DH PVA, the greatest absorbance was found in the sample that was treated for 4 h, whilst in the case of the 99% DH PVA, this occurred for the one treated for 8 h. On day 7, 1 h heat-treated materials achieved little proliferation for both sample series, whilst all other materials showed clear cell proliferation, with 4 h having the greatest average proliferation for both grades of PVA. Notably, mats treated for 16 h began to exhibit poor wettability, which resulted in highly variable results, as indicated by the substantial error bars on the graphs. Overall, there did not appear to be a notable difference in biocompatibility between the 98% and 99% hydrolysed samples based on the CCK-8 assay.

**Figure 9:**
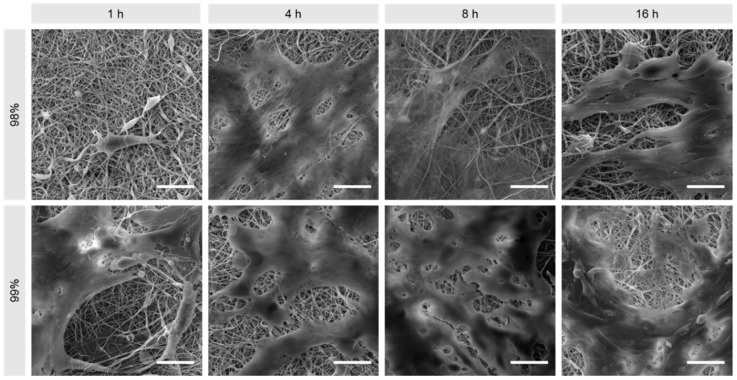
Representative SEM images of 3T3 cells on 98% and 99% DH PVA mats produced by electrospinning 7 days after cell seeding. Scale bars: 20 µm.

To validate the apparent cytocompatibility of the electrospun nanofibres and confirm cell adhesion and proliferation, day 7 samples had their cells fixed using GA and were imaged by SEM (Figure 9). Those treated for 4 and 8 h exhibit a relatively smooth cell monolayer with somewhat uniform coverage, whilst the ones treated for 16 h formed clusters of cells that did not spread evenly along the surface of the mats.

#### 3.3.2. Thrombogenicity

**Figure 10:**
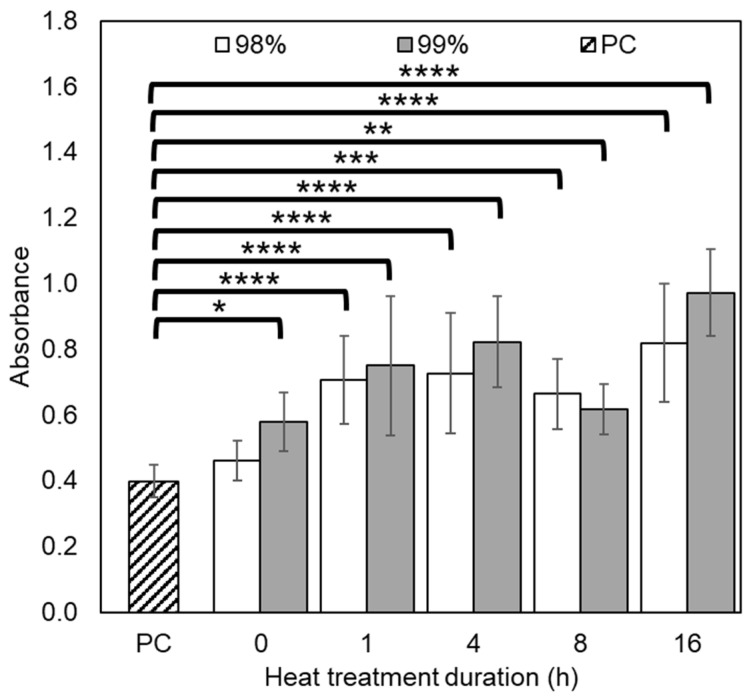
Cell viability results of thrombocyte activity on 98% and 99% DH PVA mats following heat treatment and 2 h exposure to TRS, with absorbance measured at 450 nm. Key: positive control/PC—well plate plastic (cross-hatched); 98% (white); 99% (grey). * *p* ≤ 0.05, ** *p* ≤ 0.01, *** *p* ≤ 0.001, **** *p* ≤ 0.0001.

Materials were exposed to TRS for 2 h, rinsed and then measured for thrombogenicity using a CCK-8 assay, wherein viability corresponds to rate of platelet activation. Figure 10 shows that all samples had an increase in thrombogenicity relative to the positive control, though this effect was less pronounced in the untreated and 8 h treated fibres. Overall, however, there appears to be a positive trend correlating thrombogenicity and heat treatment duration for both materials (*p* < 0.0001), with DH also playing a role in thrombogenicity (*p* = 0.0108). Given that in general the fibrous structure of electrospun materials is a thrombogenic factor, the stabilising effect of heat treatment on the material morphology predictably increased thrombogenicity, with the exception of the samples treated for 8 h, which is a paradoxical effect and difficult to explain [28]. Conversely, it is suggested that the lack of thermal stabilisation is the reason for lower thrombogenicity in untreated samples, as the fibrous structure was not preserved due to the dissolution and formation of a hydrogel and therefore could not act as a thrombogenic factor.

**Figure 11:**
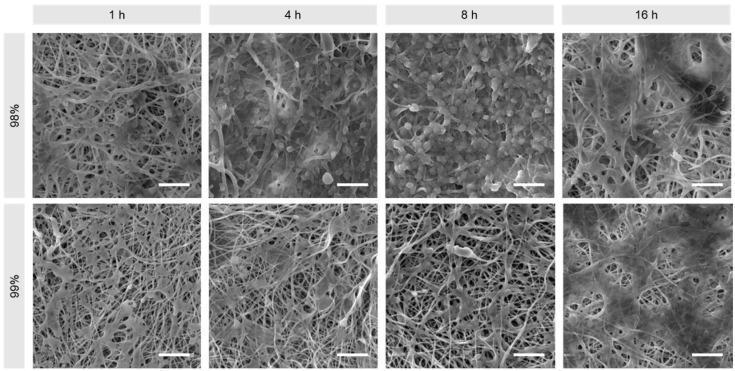
Representative SEM images of activated platelets on 98% and 99% DH PVA mats produced by needleless electrospinning. Scale bars: 10 µm.

Examination of the materials under SEM (Figure 11) reveals the presence of activated platelets on all samples, largely forming a smooth layer and bridging between fibres. In the case of the 8 h treated samples, the thrombocytes seem to be less spread out, withdrawn, and potentially detaching or inadequately attached.

#### 3.3.3. Coagulation

Adsorption of proteins from blood plasma to the material surface can cause changes in the rate of coagulation. The APTT (intrinsic pathway) and PT (extrinsic pathway) were performed to test if the materials have any anticoagulation effect or cause accelerated coagulation, with the normal bounds being 23–35 s for APTT and 10–15 s for PT [28]. Clinical plasma in the absence of any materials was used as a test control.

**Figure 12:**
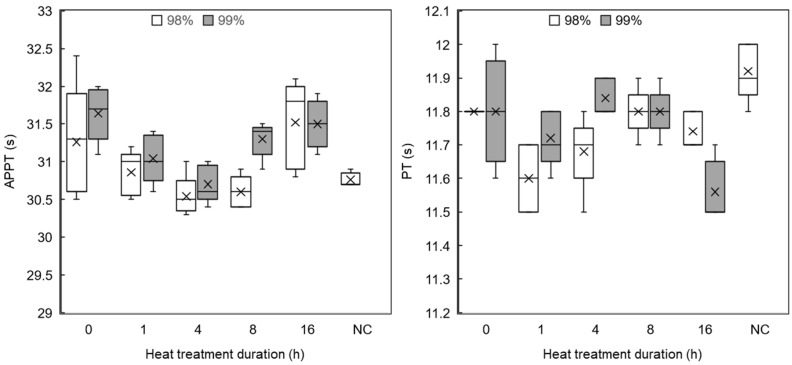
Box and whisker plots of APTT (**left**) and PT (**right**) coagulation tests of nanofibrous mats produced from 98% and 99% hydrolysed PVA after heat treatment. Key: 98% (white); 99% (grey). Control/NC—clinical plasma in isolation (n = 5).

The APTT test, shown in Figure 12 (left-hand side), demonstrated that samples produced from 99% DH PVA tended to have slight anticoagulation effects compared to 98% DH samples, but on average, all samples were ≤3% above the negative control. The PT test (Figure 12, right-hand side) showed that all materials from both sample series had a minor accelerating effect on coagulation time, but once again, the difference was ≤3%. Though all samples exhibited some deviation from the control, it was not considered to be substantial in a clinical context [49].

#### 3.3.4. Haemolysis

Haemolysis can be caused when interaction between erythrocytes and a given material may lead to damage to the red blood cell membrane, resulting in leakage of haemoglobin into solution and potentially eliciting entire disintegration of the cells. Haemolysis absorbance values measured at 570 nm can be normalised against positive controls (erythrocytes in contact with distilled water), in order to plot haemolysis as a percentage rather than as absorbance values. Haemolysis testing is governed by ISO 10993 [50], which states that blood-contacting medical devices should not exhibit >5% haemolysis.

**Figure 13:**
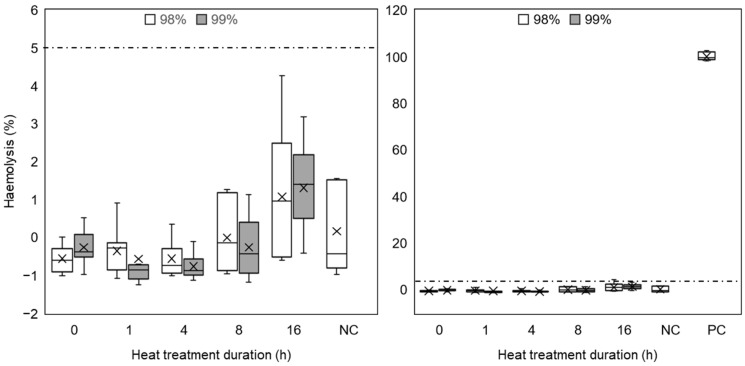
Haemolysis percentage of red blood cells after incubation with nanofibrous materials. Absorbance at 570 nm measures haemoglobin released in the solution after centrifugal separation of erythrocytes. Absorbance values for controls were measured for distilled water (PC) and PBS (NC) and then all values were normalised against the positive control. The left-hand-side graph excludes PC to allow focusing on the area of interest, while the right-hand-side graph has the PC included. Dashed line indicates 5% haemolysis. Key: 98% DH (white); 99% DH (grey) (n = 5).

Figure 13 shows haemolysis results for 98% and 99% DH PVA electrospun materials after heat treatment, along with positive and negative controls. Average haemolysis values were almost equal for both species of PVA at equivalent heat treatment durations, showing a gradual increase as the treatment duration progressed. All materials, other than those subject to 16 h of heat treatment, had lower average haemolysis values than the negative control (erythrocytes in PBS), and even those at 16 h did not exceed the 5% threshold of haemolysis laid out by the governing standard (indicated by the dashed line), and begun to exhibit greater variability in data, which is consistent with the biocompatibility results (Section 3.3.1). 

**Figure 14:**
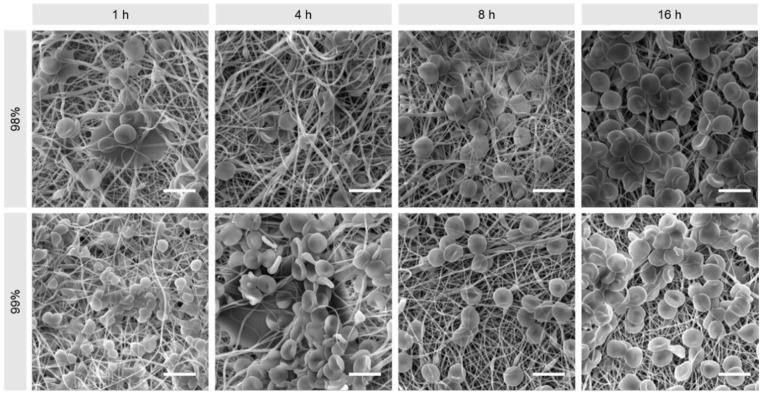
SEM images of nanofibrous materials produced by needless electrospinning after fixing of red blood cells. Scale bars: 10 µm.

Figure 14 shows SEM images of erythrocytes fixed upon the nanofibrous materials. The untreated samples are not included because they dissolved, and it was not possible to recover them from the solution following the assay. Where haemolysis has occurred, it is typically expected to observe evidence of crenation of the cells due to membrane damage. However, crenated or otherwise damaged cells were not evident on any samples, regardless of PVA species or heat treatment duration, which is a very encouraging result for many TE applications.

## 4. Conclusions

Electrospinning PVA with differing degrees of hydrolysis (DH) followed by heat treatment at durations between 0 and 16 h has the potential to create almost bead-free non-woven nanofibrous supports, with properties that can be tailored for various tissue engineering (TE) applications. The DH influences fibre diameter, and the heat treatment process at 180 °C produces some chemical changes that have implications on the hydrophilicity of the sample, as demonstrated by contact angle measurements. 

Biocompatibility testing of all materials revealed a bell-shaped behaviour between 1 and 16 h of heat treatment duration, with 4 h corresponding to the greatest cell proliferation after 7 days. The haemocompatibility assay indicated that while neither degree of hydrolysis nor treatment duration had a notable effect on coagulation time or haemolysis when in contact with blood, some thrombogenic effect was observed for samples treated for 1, 4 and 16 h. Combined, the results suggest that PVA electrospun materials that thermally stabilised for upward of 4 h show promise for TE applications, with the possible exception of situations involving long-term blood contact, such as vascular TE, where thrombin formation is a concern.

An obvious limitation of this work is the use of only one temperature for support stabilisation. Further investigation involving a range of temperatures, combined with various heat treatment durations, can help establish the possible mechanism that causes a decrease in PVA solubility due to heat treatment and is currently underway in our laboratories.

## Figures and Tables

**Table 1:** Crystallinity of nanofibrous samples after heat treatment calculated from XRD data.

Heat Treatment Duration (h)	Crystallinity	
	98% DH PVA	99% DH PVA
0	19.9	39.0
1	29.2	53.4
4	31.1	53.6
8	26.8	57.0
16	19.5	19.5

## Data Availability

Data are contained within the article or Supplementary Materials.

## Supplementary Materials

The following supporting information can be downloaded at: https://www.mdpi.com/article/10.3390/polym16142079/s1, Figure S1: Histograms of fibre diameters of electrospun PVA samples produced by DC Needleless electrospinning, following 0–16 h heat treatment duration at 180 °C; Figure S2: Stress-strain curves of electrospun PVA samples produced by DC Needleless electrospinning, following 0–16 h heat treatment duration at 180 °C; Figure S3: Representative images from sessile drop contact angle testing of films produced from 98% and 99% hydrolysed PVA films after thermal stabilization; Table S1: Summary of peaks commonly associated with FT-IR spectrum of PVA.
