# Supplementary material for: Thermally Stabilised Poly(vinyl alcohol) Nanofibrous Materials Produced by Scalable Electrospinning: Applications in Tissue Engineering
Source: Polymers (Basel). 2024 Jul 21;16(14):2079. doi: 10.3390/polym16142079 (PMC11281220; doi:10.3390/polym16142079)
Supplement: Supplementary file 1 [file polymers-16-02079-s001.zip › polymers-3059477-supplementary.pdf]

Electronic Supplementary Information

Thermally stabilised poly(vinyl alcohol) nanofibrous materials produced by scalable electrospinning: Applications in tissue engineering

W. Joseph A. Homer[a,b], Maxim Lisnenko[c], Sarka Hauzerova[c], Bohdana Heczkova[d], Adrian C. Gardner[e,f], Eva K. Kostakova[c], Paul D. Topham[b,g], Vera Jencova[c], Eirini Theodosiou[a,b,*]

[a] Engineering for Health Research Centre, College of Engineering and Physical Sciences, Aston University, Birmingham, UK
[b] Chemical Engineering and Applied Chemistry, College of Engineering and Physical Sciences, Aston University, Birmingham, UK
[c] Department of Chemistry, Faculty of Science, Humanities and Education, Technical University of Liberec, Liberec, Czech Republic
[d] Department of Haematology, Regional Hospital Liberec, Liberec, Czech Republic
[e] The Royal Orthopaedic Hospital NHS Foundation Trust, Birmingham, UK
[f] College of Health and Life Sciences, Aston University, Birmingham, UK
[g] Advanced Materials Research Centre, Aston University, Birmingham, UK

Table S1. Summary of peaks commonly associated with FT-IR spectrum of PVA [1-3].

Peak absorption (cm^{-1})	Group
**3000-3500**	O-H stretching
**2900-2950**	C-H stretching
**1710-1750**	C=O stretching
**1600-1650**	C=C stretching
**1410**	C-H vibration
**1330**	C-O vibration
**1140**	C-O stretching
**1096**	O-H bending

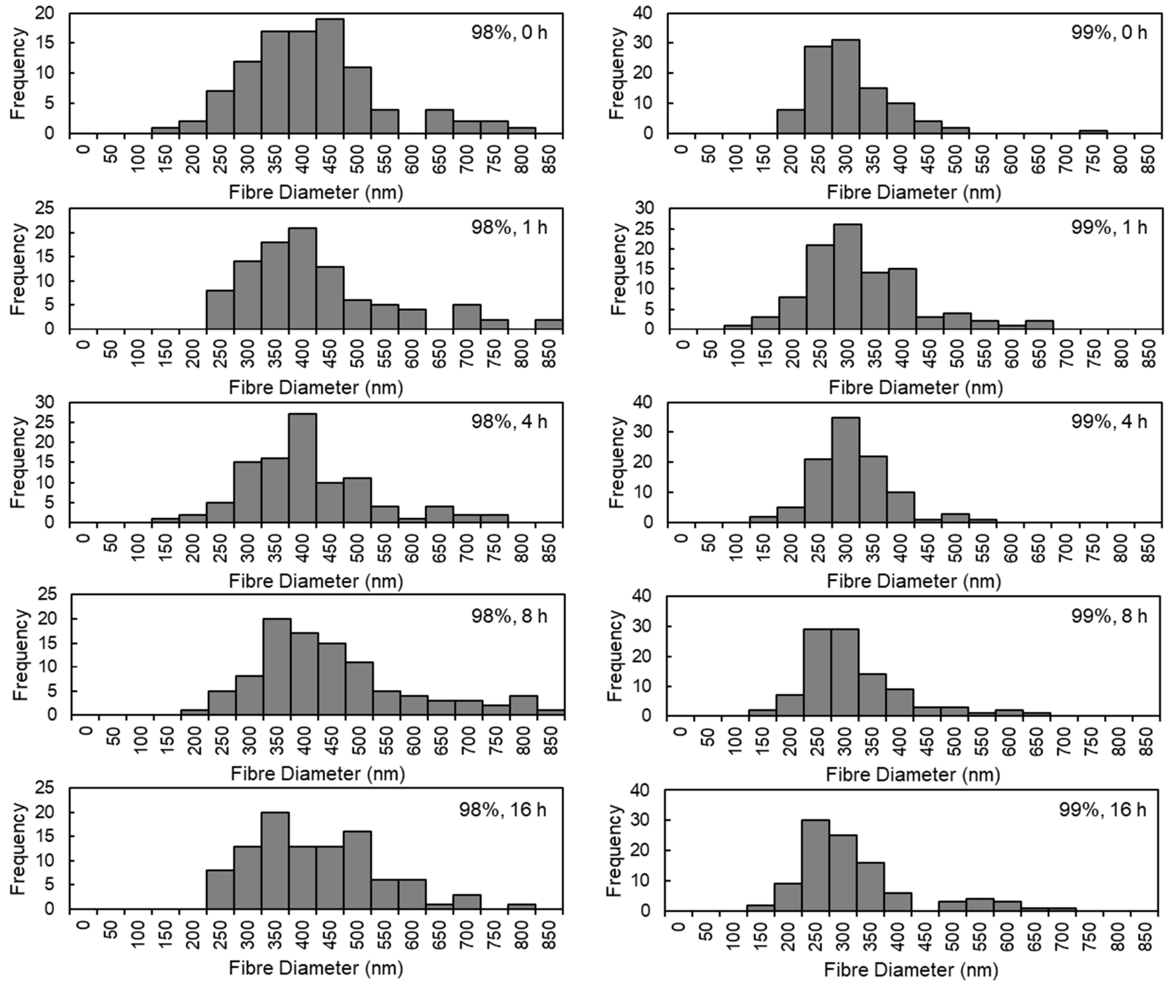

Figure S1. Histograms of fibre diameters of electrospun PVA samples produced by DC Needleless electrospinning, following 0-16 h heat treatment duration at 180 °C.

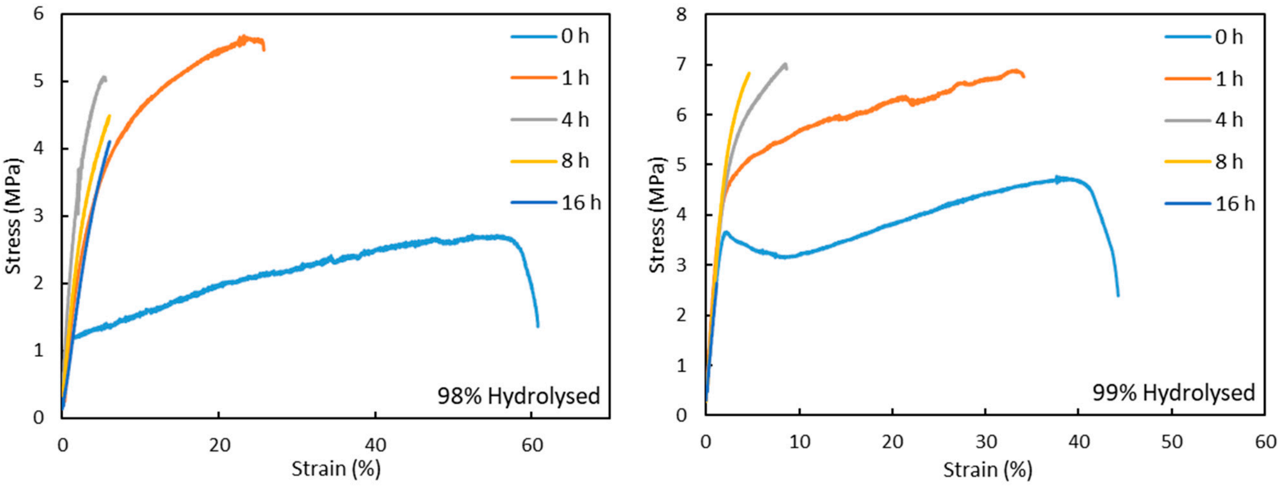

Figure S2. Stress-strain curves of electrospun PVA samples produced by DC Needleless electrospinning, following 0-16 h heat treatment duration at 180 °C.

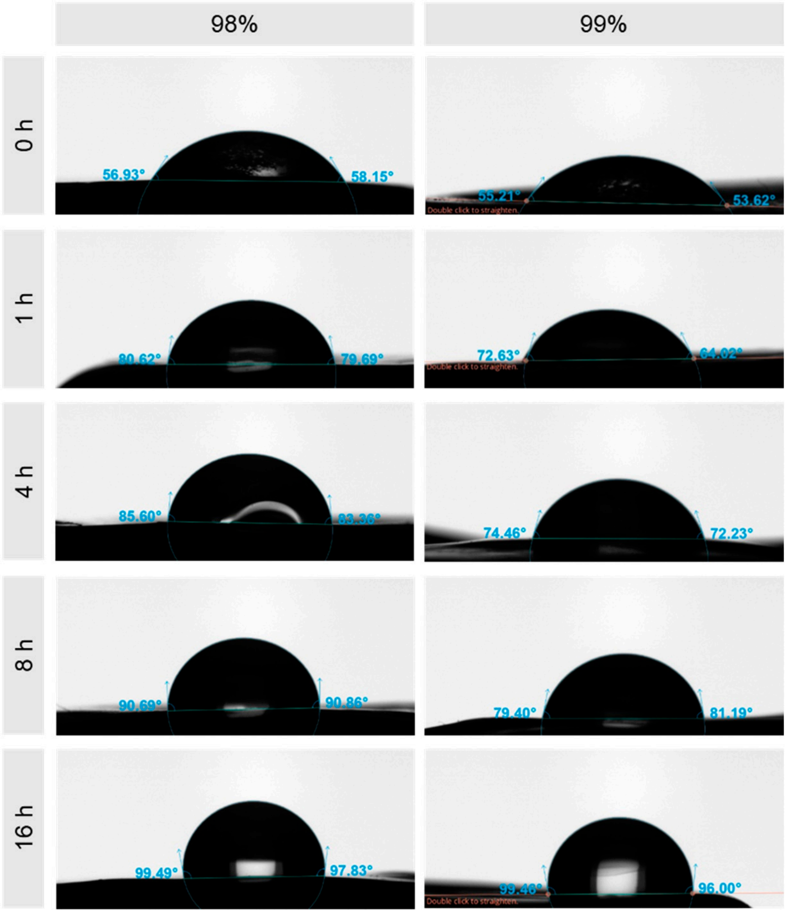

Figure S3. Representative images from sessile drop contact angle testing of films produced from 98% and 99% hydrolysed PVA films after thermal stabilisation.

References

1. Tretinnikov, O.N. and S.A. Zagorskaya, *Determination of the degree of crystallinity of poly(vinyl alcohol) by FT-IR spectroscopy.* Journal of Applied Spectroscopy, 2012. **79**(4): p. 521-526.
2. Alhosseini, S.N., et al., *Synthesis and characterization of electrospun polyvinyl alcohol nanofibrous scaffolds modified by blending with chitosan for neural tissue engineering.* International Journal of Nanomedicine, 2012. **7**: p. 25-34.
3. Jipa, I.M., et al., *Effect of gamma irradiation on biopolymer composite films of poly(vinyl alcohol) and bacterial cellulose.* Nuclear Instruments & Methods in Physics Research Section B-Beam Interactions with Materials and Atoms, 2012. **278**: p. 82-87.
